# Consumption Patterns and Product Format Preferences of Inner Beauty Functional Foods Among Korean Adults

**DOI:** 10.3390/foods15101820

**Published:** 2026-05-21

**Authors:** Eunjeong Park, Ki Han Kwon

**Affiliations:** 1 Division of Beauty Arts Care, Department of Practical Arts, Graduate School of Culture and Arts, Dongguk University, Seoul 04620, Republic of Korea; 2 College of General Education, Kookmin University, Seoul 02707, Republic of Korea

**Keywords:** inner beauty, functional food, consumption patterns, product format, Korean consumers, food labeling

## Abstract

The inner beauty functional food sector has grown rapidly in South Korea. These products are orally consumed bioactive formulations designed to improve skin health, hair vitality, and overall wellness. However, empirical evidence on consumption patterns and product format preferences across different demographic groups remains limited. This cross-sectional study examined consumption patterns, purchase channels, and product format preferences among 502 Korean adults who had experience with inner beauty functional foods. Chi-square analysis was used to examine differences in consumption reasons, duration of use, purchase channels, and product format preferences according to socio-demographic characteristics. Results showed that skin health was the dominant consumption motivation (47.6%), particularly among younger and female consumers, while weight management and hair and nail health were more prevalent among older adults. Online purchasing dominated (57.8%), with significant age- and education-based variation; consumers in their 20s purchased online at 67.5%, declining to 44.4% among those aged 40 and above. Capsule and tablet formats were most prevalent overall (41.6%), with males, married consumers, and graduate-degree holders showing significantly stronger preference for this format, whereas gummy and chewable formats were more frequently preferred by female consumers. These findings provide practical implications for inner beauty producers, food distributors, and nutrition educators seeking to align product development and communication strategies with the heterogeneous preferences of Korean inner beauty consumers.

## 1. Introduction

Inner beauty functional foods are orally consumed products designed to improve skin condition, hair quality, nail health, and overall wellness [1,2]. These products have become one of the fastest-growing sectors in the global nutraceutical market [3,4,5]. Asia, particularly Japan and South Korea, has been a major driver of global demand for inner beauty products, accounting for nearly 70% of the global beauty supplement market value [6]. In South Korea specifically, the health functional food (HFF) market reached KRW 5.21 trillion in 2023, with a five-year compound annual growth rate of 9.8%, and skin health consistently ranked as a top motivator for supplement consumption across demographic groups [7,8]. Despite rapid market growth, research on inner beauty functional food consumption in South Korea remains limited. Most available studies address general functional food acceptance [9,10,11], consumer trust in health claims [12,13,14], or willingness to pay for specific ingredients such as collagen peptides, without characterizing the actual consumption patterns [15,16,17], product format preferences, and purchase channel choices that differentiate consumer subgroups [18,19]. Understanding how demographic factors influence consumption behavior is important for product development and nutrition education strategies [20,21]. Food labeling and product format characteristics function as credence attributes—qualities that consumers cannot verify through direct inspection and therefore rely on as informational signals to guide purchase decisions [22,23,24]. Research across functional food categories confirms that product format conveys implicit signals about efficacy, convenience, and legitimacy: capsule and tablet formats are commonly associated with clinical efficacy, while gummy and chewable formats are valued for palatability and ease of consumption, especially among younger demographics [25]. The divergence in format preference across age, sex, and education groups has been documented in the broader dietary supplement literature but has not been systematically examined within the inner beauty subcategory in South Korea. Purchase channel selection also varies according to demographic characteristics.

In South Korea, e-commerce accounts for approximately 68% of health functional food sales, particularly among consumers in their 30s, while older and lower-income consumers continue to rely on physical retail channels including pharmacies and large supermarkets [7,26]. Understanding how channel preferences differ across demographic groups provides useful insights for omnichannel distribution planning [27,28].

Against this background, the present study aimed to (1) describe inner beauty functional food consumption patterns including consumption reasons, duration of use, purchase channel selection, and product format preferences among Korean adults; and (2) determine how socio-demographic characteristics differentiate these patterns through chi-square analysis. The findings are intended to inform product development, distribution strategy, and nutrition education programming for the Korean inner beauty market.

## 2. Materials and Methods

### 2.1. Study Design and Participants

A cross-sectional online survey was conducted among Korean adults who had consumed inner beauty functional foods within the previous 12 months. Participants were recruited through a professional online survey panel between 15 January and 20 January 2026. Participants were eligible if they: (1) were Korean adults aged 20 years or older and (2) had consumed inner beauty functional foods at least once during the previous year. After exclusion of incomplete responses, 502 valid cases were retained. Ethical review and approval were waived for this study in accordance with Article 15 (2) of the Bioethics and Safety Act and Article 13 of its Enforcement Rules (Ministry of Health and Welfare, Republic of Korea), as this study involved an anonymous online survey and did not collect any personally identifiable or sensitive information.

### 2.2. Survey Instrument

The structured questionnaire collected data across five domains: (1) socio-demographic characteristics (sex, age, occupation, monthly household income, education, marital status, and region of residence); (2) primary reason for inner beauty product consumption (skin health, weight management, hair and nail health, immunity/fatigue improvement); (3) duration of consumption (less than 1 month; 1 to less than 6 months; 6 months to less than 1 year; 1 year or more); (4) primary purchase channel (online, pharmacy/drugstore, large supermarket/department store, acquaintance/word-of-mouth referral); and (5) preferred product format (capsule/tablet, gummy/chewable, powder, beverage). Examples of inner beauty functional foods, including collagen supplements, hyaluronic acid products, and beauty-oriented dietary supplements, were provided within the survey to ensure consistent participant understanding.

### 2.3. Statistical Analysis

Data were analyzed using SPSS WIN 28.0. Frequency analysis described distributions of all categorical variables. Chi-square (χ^2^) tests examined associations between socio-demographic characteristics and consumption-related outcomes. Significance was evaluated at *p* < 0.05 for all tests.

## 3. Results

### 3.1. Participant Characteristics

The sample comprised 502 Korean adults. The majority were female (84.9%), aged in their 30s (55.2%), and employed as office workers (68.7%). Most held a bachelor’s degree (71.7%) and were unmarried (77.3%). The most common monthly income bracket was KRW 3–4 million (43.0%). Full socio-demographic characteristics are presented in Table 1.

### 3.2. Consumption Patterns

**Figure 1:**
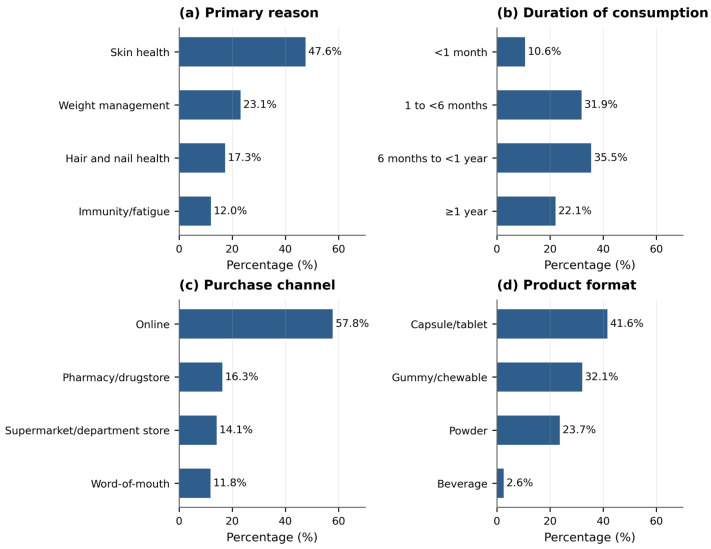
Overall consumption patterns of inner beauty functional foods among Korean adults (*n* = 502). (**a**) Primary reason for consumption; (**b**) duration of consumption; (**c**) primary purchase channel; (**d**) preferred product format.

Skin health was the most frequently reported reason for inner beauty product consumption (47.6%), followed by weight management (23.1%), hair and nail health (17.3%), and immunity/fatigue improvement (12.0%). The most common duration of use was 6 months to less than 1 year (35.5%), followed by 1 to less than 6 months (31.9%), and 1 year or more (22.1%). Online channels accounted for 57.8% of purchases, followed by pharmacy/drugstore (16.3%), large supermarket/department store (14.1%), and acquaintance/word-of-mouth (11.8%). Capsule/tablet was the most frequently preferred product format (41.6%), followed by gummy/chewable (32.1%), powder (23.7%), and beverage (2.6%). Full consumption and usage patterns are provided in Table 2 and Figure 1.

### 3.3. Socio-Demographic Differences in Consumption Reason

Chi-square analysis showed significant differences in consumption reasons according to socio-demographic characteristics (Table 3). Female participants were more likely than male participants to report skin health as their primary reason for consumption (49.3% vs. 38.2%; χ^2^ = 13.950, *p* = 0.003). Age was significantly associated with consumption reason (χ^2^ = 29.792, *p* < 0.001): skin health motivation was highest among consumers in their 20s (54.0%) and declined with age (40 and above: 36.4%), while weight management motivation increased progressively with age. Significant occupation-based differences were also observed (χ^2^ = 35.032, *p* < 0.001), with university students reporting the highest skin health motivation (58.1%) and professionals the lowest (27.6%). Education level was a significant predictor (χ^2^ = 17.630, *p* = 0.040), and regional differences were significant (χ^2^ = 26.570, *p* = 0.009), with Seoul/Gyeonggi-do residents more frequently citing skin health (52.2%) compared to Jeolla region residents (30.0%).

### 3.4. Socio-Demographic Differences in Purchase Channel

**Figure 2:**
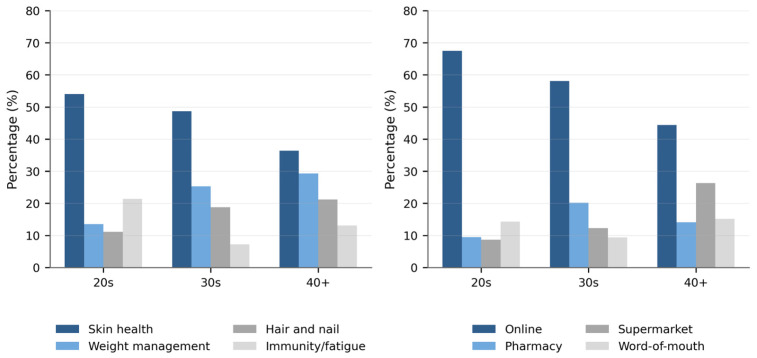
Age-group differences in primary consumption reason and purchase channel preference among Korean inner beauty functional food consumers (*n* = 502).

Table 4 presents chi-square results for purchase channel preference. Sex was a significant predictor (χ^2^ = 12.710, *p* = 0.005): females used pharmacies/drugstores at substantially higher rates than males (18.5% vs. 3.9%), while online purchase rates were similar between sexes. Age significantly influenced channel selection (χ^2^ = 28.079, *p* < 0.001): online purchasing declined from 67.5% among consumers in their 20s to 44.4% among those aged 40 and above, while supermarket purchasing increased with age. Education (χ^2^ = 24.959, *p* = 0.003) and marital status (χ^2^ = 13.152, *p* = 0.004) were also significant, with higher-educated and single consumers more frequently purchasing online. Region was also significantly associated with purchase channel preference (χ^2^ = 22.028, *p* = 0.037), with Seoul/Gyeonggi-do residents showing the highest online purchase rate (62.3%). Age-related differences in both consumption reason and purchase channel are illustrated in Figure 2.

### 3.5. Socio-Demographic Differences in Product Format Preference

**Figure 3:**
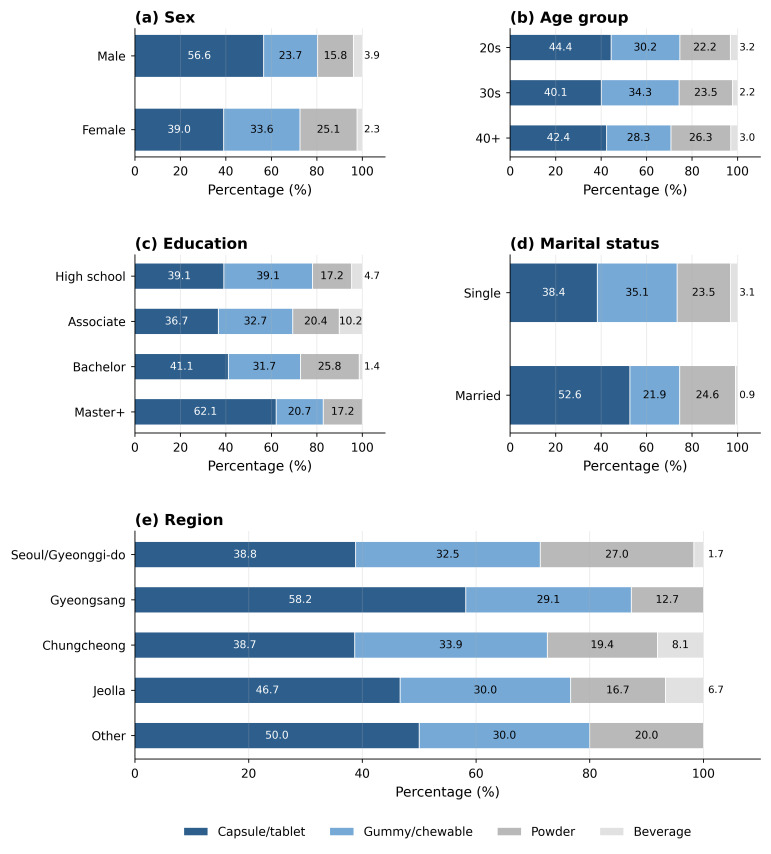
Product format preference by sex, age group, education level, marital status, and region among Korean inner beauty functional food consumers ( *n* = 502).

Product format preferences differed significantly across sex, education, marital status, and region, whereas age group was not significantly associated with product format preference (Table 5 and Figure 3). Sex was a significant predictor (χ^2^ = 9.774, *p* = 0.021): males showed stronger preference for capsule/tablet formats (56.6%) while females more frequently selected gummy/chewable options (33.6%). Education was a significant predictor (χ^2^ = 22.871, *p* = 0.006): graduate-degree holders showed a higher preference for capsule/tablet formats (62.1%) compared to associate degree holders (36.7%). Married consumers preferred capsule/tablet formats at higher rates than single consumers (52.6% vs. 38.4%; χ^2^ = 10.731, *p* = 0.013). Region was also significantly associated with product format preference (χ^2^ = 22.290, *p* = 0.034), with Gyeongsang residents showing the highest capsule/tablet preference (58.2%).

## 4. Discussion

This study provides a detailed empirical profile of inner beauty functional food consumption patterns and product format preferences among Korean adults, extending the limited existing literature on this rapidly growing market segment [29,30]. Skin health was the most common reason for consuming inner beauty functional foods (47.6%). This finding is consistent with previous studies showing that women aged 25–44 are the main consumers of beauty supplements [6]. However, the significant age-based stratification identified here adds important nuance: skin health motivation was concentrated among consumers in their 20s, while 40-and-above consumers showed a more distributed motivation profile encompassing weight management and hair and nail health. Consumption goals differed across age groups. Younger consumers focused more on skin health, whereas older consumers showed greater interest in weight management and hair and nail health. These findings are consistent with previous studies showing that health priorities change across the life course [31,32]. The strong emphasis on skin health among consumers in their 20s may reflect the increasing influence of the appearance-oriented wellness culture and preventive beauty-management behaviors among younger Korean consumers [33,34,35,36]. These findings suggest that inner beauty producers should avoid uniform product messaging and instead tailor benefit claims according to age-specific health and beauty priorities [37,38].

Online purchasing was the dominant purchasing channel (57.8%), which aligns with documented Korean HFF market trends showing the strong role of e-commerce in this market [7]. Online purchase frequency declined with age, from 67.5% among consumers in their 20s to 44.4% among those aged 40 and above. This pattern may reflect generational differences in digital commerce literacy documented in the general functional food literature [26]. Because inner beauty products are credence-oriented products whose efficacy cannot be directly verified at the time of purchase, consumers may rely on purchase channels as signals of product reliability and safety [39,40]. In addition, the higher pharmacy utilization among female consumers (18.5% vs. 3.9% for males) may reflect gender-specific product evaluation behaviors, as female consumers may more frequently seek in-person consultation and ingredient verification before purchasing inner beauty products. This supports the interpretation that pharmacy channels function as risk-reduction spaces, offering perceived professional credibility and opportunities for ingredient verification prior to purchase [39,41]. These findings suggest that omnichannel strategies combining online platforms with pharmacy-based distribution may be effective for reaching diverse consumer groups [42,43].

The findings on product format preferences provide practical implications for product development and marketing strategies [44,45,46]. Product format preferences differed significantly by sex, education level, marital status, and region. In particular, the higher preference for capsule/tablet formats among graduate-degree holders may be consistent with prior nutraceutical research suggesting that higher health literacy is associated with preference for formats with stronger clinical connotations [19,47]. Capsule and tablet formats may convey stronger perceptions of scientific legitimacy and therapeutic efficacy, particularly among highly educated consumers with greater health literacy [48]. Conversely, the higher gummy/chewable preference among female consumers reflects the ingestible beauty industry’s broader trend toward palatability-centered delivery formats, which have rapidly gained market share globally [6]. In contrast, gummy and chewable formats may reduce psychological barriers to supplement intake by increasing sensory enjoyment and convenience among younger consumers [48,49]. The significant preference for capsule/tablet formats among graduate-degree holders (62.1%) versus associate degree holders (36.7%) suggests that education-related differences in health product literacy translate directly into format preferences [50]. This finding has direct implications for how producers position and label products targeting different educational segments.

The regional variation in consumption motivation—with Seoul/Gyeonggi-do residents more frequently citing skin health and Jeolla region residents more frequently citing weight management—may reflect the influence of regional food culture, dietary exposure to specific product marketing, and demographic composition differences across Korean administrative regions [51]. Regional variation may also reflect differences in urbanization, retail accessibility, and exposure to beauty-related marketing environments [52,53]. These regional differences suggest the need for geographically differentiated marketing and distribution strategies for inner beauty producers serving diverse Korean regional markets [54,55].

Several limitations should be acknowledged. First, the online sampling methodology, while efficient, may underrepresent older and rural consumers with limited digital access. In addition, the sample was disproportionately composed of female, relatively younger, and highly educated participants, which may limit the generalizability of the findings to the broader Korean adult population. Second, consumption duration and purchase frequency data were self-reported and subject to recall bias. Third, the study did not capture information on specific product ingredients or brand preferences, limiting the precision of product format preference analysis. Fourth, the cross-sectional design precludes causal inference regarding demographic determinants of consumption patterns. Future research should employ longitudinal designs to track consumption pattern evolution across life-stage transitions, incorporate objective purchase records to validate self-reported channel and format data, and extend the analysis to rural and lower-income consumer segments currently underrepresented in the inner beauty literature. Future studies should also investigate ingredient-specific preferences and longitudinal changes in inner beauty consumption behavior across different life stages [35,38].

## 5. Conclusions

This study provides a comprehensive empirical characterization of inner beauty functional food consumption patterns and product format preferences among Korean adults. Skin health was the dominant consumption motivation, with significant age- and sex-based variation in motivation distribution. Online purchasing dominated across all demographic groups but showed a significant age-related decline. Product format preferences were meaningfully differentiated by sex, education, marital status, and region, with capsule/tablet formats preferred by males, more highly educated consumers, and married consumers, and gummy/chewable formats favored by female consumers.

For inner beauty producers and distributors, these findings suggest that product development and marketing strategies should be tailored according to demographic characteristics. Differentiated format portfolios that serve both the clinical-efficacy-oriented preferences of graduate consumers and the palatability-driven preferences of female consumers represent a viable product development direction. Omnichannel distribution that sustains offline pharmacy presence for female consumers while prioritizing digital platforms for younger demographics can maximize market reach across the diverse Korean inner beauty consumer landscape. Future studies should further investigate longitudinal changes in inner beauty consumption behavior and explore how evolving digital health communication environments influence consumer preferences across different demographic groups.

## Figures and Tables

**Table 1:** Socio-demographic characteristics of participants (*n* = 502).

Variable	*n*	%
Sex		
Male	76	15.1
Female	426	84.9
Age group		
20s	126	25.1
30s	277	55.2
40 and above	99	19.7
Occupation		
Office worker	345	68.7
University student	62	12.4
Self-employed	36	7.2
Full-time homemaker	30	6.0
Professional	29	5.8
Monthly household income (KRW)		
<KRW 1 million	56	11.2
KRW 1–2 million	34	6.8
KRW 2–3 million	152	30.3
KRW 3–4 million	216	43.0
≥KRW 4 million	44	8.8
Education		
High school graduate	64	12.7
Associate degree	49	9.8
Bachelor’s degree	360	71.7
Master’s degree or above	29	5.8
Marital status		
Single	388	77.3
Married	114	22.7
Total	502	100.0

KRW = South Korean Won.

**Table 2:** Inner beauty functional food consumption patterns (*n* = 502).

Variable	*n*	%
Primary reason for consumption		
Skin health	239	47.6
Weight management	116	23.1
Hair and nail health	87	17.3
Immunity/fatigue improvement	60	12.0
Duration of consumption		
Less than 1 month	53	10.6
1 to less than 6 months	160	31.9
6 months to less than 1 year	178	35.5
1 year or more	111	22.1
Primary purchase channel		
Online	290	57.8
Pharmacy/drugstore	82	16.3
Large supermarket/department store	71	14.1
Acquaintance/word-of-mouth referral	59	11.8
Product format		
Capsule/tablet	209	41.6
Gummy/chewable	161	32.1
Powder	119	23.7
Beverage	13	2.6
Total	502	100.0

**Table 3:** Chi-square analysis of primary consumption reason by socio-demographic characteristics.

Variable	Skin Health	Weight mgmt.	Hair & Nail	Immunity	χ^2^	*p*	Cramer’s V
Sex							
Male	38.2%	26.3%	11.8%	23.7%	13.950	0.003 **	0.167 (small)
Female	49.3%	22.5%	18.3%	9.9%			
Age group							
20s	54.0%	13.5%	11.1%	21.4%	29.792	<0.001 ***	0.172 (small)
30s	48.7%	25.3%	18.8%	7.2%			
40 and above	36.4%	29.3%	21.2%	13.1%			
Occupation							
Office worker	49.0%	24.1%	18.0%	9.0%	35.032	<0.001 ***	0.153 (small)
University student	58.1%	14.5%	11.3%	16.1%			
Professional	27.6%	20.7%	31.0%	20.7%			
Education							
High school graduate	50.0%	28.1%	12.5%	9.4%	17.630	0.040 *	0.108 (small)
Master’s or above	55.2%	6.9%	17.2%	20.7%			
Region							
Seoul/Gyeonggi-do	52.2%	21.4%	15.4%	11.0%	26.570	0.009 **	0.133 (small)
Jeolla	30.0%	43.3%	6.7%	20.0%			

* *p* < 0.05; ** *p* < 0.01; *** *p* < 0.001. Abbreviated rows shown; full frequencies available upon request.

**Table 4:** Chi-square analysis of purchase channel preference by socio-demographic characteristics.

Variable	Online	Pharmacy	Supermarket	Word-of-Mouth	χ^2^	*p*	Cramer’s V
Sex							
Male	59.2%	3.9%	21.1%	15.8%	12.710	0.005 **	0.159 (small)
Female	57.5%	18.5%	12.9%	11.0%			
Age group							
20s	67.5%	9.5%	8.7%	14.3%	28.079	<0.001 ***	0.167 (small)
30s	58.1%	20.2%	12.3%	9.4%			
40 and above	44.4%	14.1%	26.3%	15.2%			
Education							
High school graduate	54.7%	9.4%	20.3%	15.6%	24.959	0.003 **	0.129 (small)
Bachelor’s degree	61.4%	16.7%	12.2%	9.7%			
Master’s or above	65.5%	13.8%	13.8%	6.9%			
Marital status							
Single	61.3%	16.5%	11.9%	10.3%	13.152	0.004 **	0.162 (small)
Married	45.6%	15.8%	21.9%	16.7%			
Region							
Seoul/Gyeonggi-do	215 (62.3%)	56 (16.2%)	39 (11.3%)	35 (10.1%)	22.028	0.037 *	0.121 (small)
Gyeongsang	30 (54.5%)	9 (16.4%)	8 (14.5%)	8 (14.5%)			
Chungcheong	27 (43.5%)	12 (19.4%)	12 (19.4%)	11 (17.7%)			
Jeolla	12 (40.0%)	5 (16.7%)	10 (33.3%)	3 (10.0%)			
Other	6 (60.0%)	0 (0.0%)	2 (20.0%)	2 (20.0%)			

* *p* < 0.05; ** *p* < 0.01; *** *p* < 0.001. Abbreviated rows shown; full frequencies available upon request.

**Table 5:** Chi-square analysis of product format preference by socio-demographic characteristics.

Variable	Capsule/Tablet	Gummy	Powder	Beverage	χ^2^	*p*	Cramer’s V
**Sex**							
Male	56.6%	23.7%	15.8%	3.9%	9.774	0.021 *	0.140 (small)
Female	39.0%	33.6%	25.1%	2.3%			
Age group							
20s	44.4%	30.2%	22.2%	3.2%	2.260	0.894	0.047 (negligible)
30s	40.1%	34.3%	23.5%	2.2%			
40 and above	42.4%	28.3%	26.3%	3.0%			
**Education**							
High school graduate	39.1%	39.1%	17.2%	4.7%	22.871	0.006 **	0.123 (small)
Associate degree	36.7%	32.7%	20.4%	10.2%			
Bachelor’s degree	41.1%	31.7%	25.8%	1.4%			
Master’s or above	62.1%	20.7%	17.2%	0.0%			
**Marital status**							
Single	38.4%	35.1%	23.5%	3.1%	10.731	0.013 *	0.146 (small)
Married	52.6%	21.9%	24.6%	0.9%			
**Region**							
Seoul/Gyeonggi-do	38.8%	32.5%	27.0%	1.7%	22.290	0.034 *	0.122 (small)
Gyeongsang	58.2%	29.1%	12.7%	0.0%			
Chungcheong	38.7%	33.9%	19.4%	8.1%			
Jeolla	46.7%	30.0%	16.7%	6.7%			
Other	50.0%	30.0%	20.0%	0.0%			

* *p* < 0.05; ** *p* < 0.01. Abbreviated rows shown; full frequencies available upon request.

## Data Availability

The data presented in this study are available on request from the corresponding author due to privacy issues.
